# Single-sample network modeling on omics data

**DOI:** 10.1186/s12915-023-01783-z

**Published:** 2023-12-29

**Authors:** Margherita De Marzio, Kimberly Glass, Marieke L. Kuijjer

**Affiliations:** 1 Channing Division of Network Medicine, Brigham and Women’s Hospital and Harvard Medicine School, Boston, MA USA; 2 Biostatistics Department, Harvard Chan School of Public Health, Boston, MA USA; 3 Centre for Molecular Medicine Norway (NCMM), Nordic EMBL Partnership, University of Oslo, Oslo, Norway

## Abstract

**Modeling networks for single samples can facilitate discoveries in biology that go beyond those found when analyzing an “aggregate” network, i.e., one that merges information across all the samples in an omics data set. In this commentary article, we discuss the history, current state, and future directions of the field of single-sample network modeling using omics data.**

## History of single-sample network inference

Biological systems are interconnected and regulated through a complex network of molecular interactions, signaling pathways, and genetic feedback loops. This complexity makes network modeling a crucial tool for understanding the underlying mechanisms driving biological and disease processes. Since the introduction of high-throughput assays, numerous methods have been developed that use omics data to model biological networks, especially gene regulatory and co-expression networks. Most such approaches harness data across multiple omics samples to construct a single network that represents the entire dataset. These “aggregate” network models have greatly contributed to our comprehension of health and disease. Nevertheless, they cannot reveal intrinsic variations in network heterogeneity at the level of the individual sample.

In contrast to many other areas in computational biology, single-sample network inference is a somewhat niche area, with only a handful of established methods; however, it is an area that is critical for relating networks to clinical features or other types of metadata, especially in heterogeneous diseases or populations, as well as large-scale datasets generated by different groups. The first attempts to extract network information for single samples worked by *layering sample-specific information* onto an existing network structure (such as a protein–protein interaction network). These methods often link gene expression information to nodes to obtain sample-specific “activity” scores for specific genes or regulators [1, 2]. Another popular approach is to select network edges or nodes with sample-specific omics information to obtain a “pruned” network for each sample. Recently, two main computational approaches, LIONESS [3] and SSN [4], have emerged that *explicitly infer single-sample networks*. Each of these methods employs a distinct mathematical framework to tackle the common challenge of inferring the network of a single sample by pooling population-level information.

## The current state of the field

LIONESS and SSN both use a leave-one-out approach, modeling a single-sample network as a linear perturbation to an aggregate population network. SSN assigns an edge to a node pair in a single “case” sample if the correlation coefficient of that pair differs significantly when the sample is added to a reference control population. The resulting output is a differential single-sample network based on correlation. Thus, SSN edges represent molecular interactions that are dysregulated in a case subject compared to a control group. Conversely, LIONESS does not distinguish between case and control samples and is designed to be applied to the output of an aggregate network inference approach. Its founding principle is that an aggregate network, summarizing biological information from the entire population, can be modeled as a linear combination of individual component networks. Based on this assumption, LIONESS generates a single-sample weighted network, wherein each edge represents the contribution of that sample to the aggregate population network. The LIONESS edge weight accounts for biological patterns that are specific to the individual as well as those shared across all the samples in the population.

LIONESS and SSN often provide the core methodology used by other single-sample network inference methods. For example, LIONESS networks have been integrated with molecular interaction databases to reconstruct refined patient-specific networks from cancer data [5]; a rescaled formulation of LIONESS has been applied to account for sample-size imbalances in dataset subpopulations [6]. Linear regression models have also been employed to identify the differential regulatory network of a case sample compared to a reference control population [7].

In addition to their broad application to transcriptomics data, single-sample network algorithms have demonstrated applicability across other omics domains, including metabolomics, epigenomics, and microbiome studies. Early applications of these algorithms generally focused on characterizing personalized cancer drivers and regulatory interactions. Over time, single-sample networks have been applied in the context of cancer, cardiovascular and respiratory diseases, and neurodevelopmental disorders and to study sex-specific differences in gene regulation. These applications have provided valuable insights into the molecular interactions that underlie individual phenotypes, advancing the field of precision medicine.

## Strengths and caveats of existing methods

Network inference approaches that are based on *layering sample-specific information* on a fixed set of known network interactions have helped identify sample-specific network properties. However, these methods, by themselves, cannot capture new interactions, which in turn need to be inferred from downstream analyses. In contrast, methods that *explicitly infer a single-sample network* do not suffer from this limitation. They also can incorporate information from additional related samples to boost sample size, thus enhancing statistical power. This can be helpful when a limited number of samples are available for a biological condition of interest.

Single-sample networks provide a distinct advantage compared to differential network approaches, since they enable comparisons when explicit groups are not available. This can, for example, be the identification of network changes associated with continuous clinical variables or the detection of new subtypes. In addition, analysis of single-sample networks can be corrected for potential confounders in a dataset, which may arise due to technical variation (batch effects) or known clinical features that contribute to network heterogeneity.

One caveat for methods that explicitly infer single-sample networks is that they work by borrowing information from a set of background samples. Therefore, single-sample networks inferred using different backgrounds may differ. Thus, it is important to carefully consider what samples to include in the background. This may be particularly challenging when dealing with heterogeneous datasets. Including samples from different groups (e.g., samples from several disease subtypes) in the background is commonly done when performing comparative network analyses between multiple groups. When the aim is to characterize networks across a homogeneous subgroup of samples, it may be best to only include samples from that subgroup, as the differences between samples may be easier to interpret.

Interpreting single-sample network edge weights can also be challenging, as the inferred weights typically do not explicitly follow the distribution of weights obtained for the aggregate network model. For example, it is unclear what “sample-specific correlation” means as single-sample edge weights inferred from correlations may not necessarily be bounded by [-1, 1]. These different distributions may impact downstream network analysis.

Finally, single-sample networks can be more sensitive to the preprocessing of the input data compared to aggregate networks, as a perturbation in a single sample is used to estimate network edges. This makes it challenging to distinguish between perturbations (and therefore edges) that are true signals compared to random noise, a problem exacerbated when data are very sparse.

## Challenges in assessing single-sample networks

One particularly challenging aspect of single-sample network inference is that there is no clear standard for systematically assessing the inferred networks. Methods often differentiate themselves by highlighting their ability to address a specific methodology issue or biological question; thus, single-sample networks have been assessed inconsistently and in ways that inevitably put the method in question in the best light.

Identifying an appropriate standard to evaluate single-sample network accuracy is non-trivial (and may be impossible). There is no set of experimentally-derived networks that can be used as a benchmark, and even if there was, it would not be appropriate in all contexts; the appropriate benchmark for single-sample correlation networks will differ from that for single-sample regulatory networks; the standard for a sample-specific differential network will differ from that for a single-sample network that captures both differential and common edges. Although simulated data may be appealing for evaluating single-sample networks, it is inherently limited. Features of data-generating models can impact covariance patterns, changing the apparent performance of methods in a manner that better reflects consistency with the generating model’s assumptions than actual network accuracy.

This begs the question of how important it is to compare the “accuracy” of single-sample network methods versus understanding their relative strengths, limitations, and potential biological applications. Unfortunately, it is nearly impossible to assess methods based solely on biological findings, which are qualitative and open to interpretation. Adding to the confusion, many single-sample network methods are often highly related mathematically, and specific aspects of them are sometimes interchangeable within larger analysis pipelines. Thus, it can be challenging to determine how the biological findings identified via downstream network analysis may differ using various approaches. Overall, it is critically important to understand what distinguishes different approaches, not only methodologically but also in terms of the biological questions each is most adept at answering.

## Future directions of the field

Current approaches that explicitly infer sample-specific networks are either specifically derived in the context of Pearson correlation (SSN) or can be applied to it (LIONESS). Due to their relationship to correlation metrics, one open question is whether it is possible to derive associated statistical errors or confidence intervals for the edges in the inferred single-sample networks. Going forward, it will also be critical to develop methods that can model additional types of biological networks that go beyond pairwise (correlation) measures. Several recent methods in this direction generate sample-specific gene regulatory networks using individual epigenetic [8] or genetic [9] profiles to modify an initial set of estimated edges. In addition, although current methods have been applied individually to various types of omics data, new single-sample inference approaches are needed that simultaneously integrate multiple times of omics data. This may be an area that is well-suited to emerging approaches that use deep learning to infer biological networks.

The recent expansion of high-resolution data types, such as single-cell and spatial omics data, will likely greatly facilitate single-sample network modeling, and several recent approaches have shifted their focus towards single-cell-specific rather than sample-specific networks [10]. Single-cell data lends itself naturally to modeling networks for single samples, which can, for example, be inferred for specific cell types. In addition, approaches that measure multi-omics data in the same cell enable the identification of direct links between various omics data types. Future directions in modeling networks based on single-cell data should address challenges with sparsity, variability in sample size, and heterogeneity and how to accurately define cell types used for network modeling.

In summary, single-sample network modeling is a relatively new field, with many remaining open questions as well as exciting opportunities for future research directions. We look forward to seeing how this field evolves over the coming years and further contributes to omics-based discoveries in biology.

## Data Availability

Not applicable.
